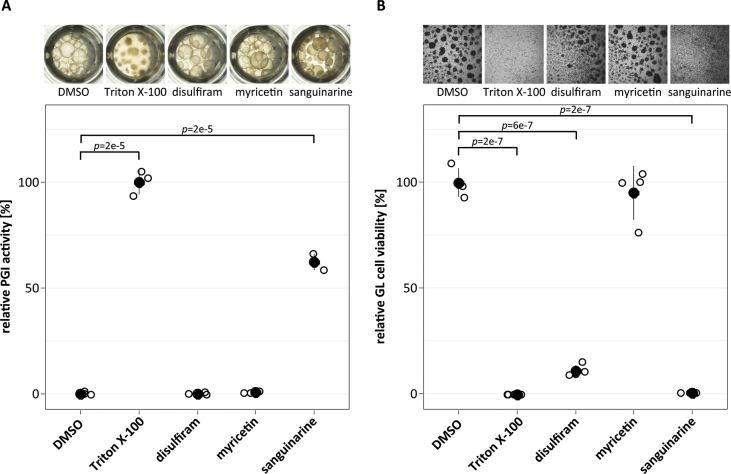# Investigation of the threonine metabolism of *Echinococcus multilocularis*: The threonine dehydrogenase as a potential drug target in alveolar echinococcosis

**DOI:** 10.1016/j.ijpddr.2025.100581

**Published:** 2025-01-18

**Authors:** Marc Kaethner, Pascal Zumstein, Joachim Müller, Matías Preza, Philipp Grossenbacher, Anissa Bartetzko, Laura Vetter, Martin Lochner, Stefan Schürch, Clement Regnault, Daniel Villalobos Ramírez, Britta Lundström-Stadelmann

**Affiliations:** a Institute of Parasitology, Vetsuisse Faculty, University of Bern, Bern, Switzerland; b Graduate School for Cellular and Biomedical Sciences, University of Bern, Bern, Switzerland; c Institute of Biochemistry and Molecular Medicine, University of Bern, Bern, Switzerland; d Department of Chemistry, Biochemistry and Pharmaceutical Sciences, University of Bern, Bern, Switzerland; e Integrated Protein Analysis - Mass Spectrometry Unit, MVLS Shared Research Facilities, College of Medical, Veterinary and Life Sciences, University of Glasgow, Glasgow, United Kingdom; f Department of Bioinformatics, University of Würzburg, Würzburg, Germany; g Multidisciplinary Center for Infectious Diseases, University of Bern, Bern, Switzerland

**Keywords:** Echinococcus multilocularis, Cestode, Threonine metabolism, Target-based screening, Disulfiram, Sanguinarine

## Abstract

Alveolar echinococcosis (AE) is a severe zoonotic disease caused by the metacestode stage of the fox tapeworm *Echinococcus multilocularis*. We recently showed that *E. multilocularis* metacestode vesicles scavenge large amounts of L-threonine from the culture medium. This motivated us to study the effect of L-threonine on the parasite and how it is metabolized. We established a novel metacestode vesicle growth assay with an automated readout, which showed that L-threonine treatment led to significantly increased parasite growth. In addition, L-threonine increased the formation of novel metacestode vesicles from primary parasite cell cultures in contrast to the non-proteinogenic threonine analog 3-hydroxynorvaline. Tracing of [U-^13^C]-L-threonine and metabolites in metacestode vesicles and culture medium resulted in the detection of [U-^13^C]-labeling in aminoacetone and glycine, indicating that L-threonine was metabolized by threonine dehydrogenase (TDH). EmTDH-mediated threonine metabolism in the *E. multilocularis* metacestode stage was further confirmed by quantitative real-time PCR, which demonstrated high expression of *emtdh* in *in vitro* cultured metacestode vesicles and also in metacestode samples obtained from infected animals. EmTDH was enzymatically active in metacestode vesicle extracts. The compounds disulfiram, myricetin, quercetin, sanguinarine, and seven quinazoline carboxamides were evaluated for their ability to inhibit recombinantly expressed EmTDH. The most potent inhibitors, albeit not very strong or highly specific, were disulfiram, myricetin and sanguinarine. These compounds were subsequently tested for activity against *E. multilocularis* metacestode vesicles and primary parasite cells and only sanguinarine demonstrated significant *in vitro* activity. However, TDH is not its only cellular target, and it is also known to be highly toxic. Our findings suggest that additional targets of sanguinarine should be explored, and that it may serve as a foundation for developing more specific compounds against the parasite. Moreover, the EmTDH assay could be a valuable high-throughput, target-based platform for discovering novel anti-echinococcal compounds.

## Highlights

• An active threonine metabolism is important for *E. multilocularis in vitro*.
• EmTDH mediates threonine catabolism in the *E. multilocularis* metacestode stage.
• An established EmTDH assay allows target-based screening for potential inhibitors.
• An automated, image-based metacestode vesicle growth assay was developed.

## Graphical abstract

**Figure:**
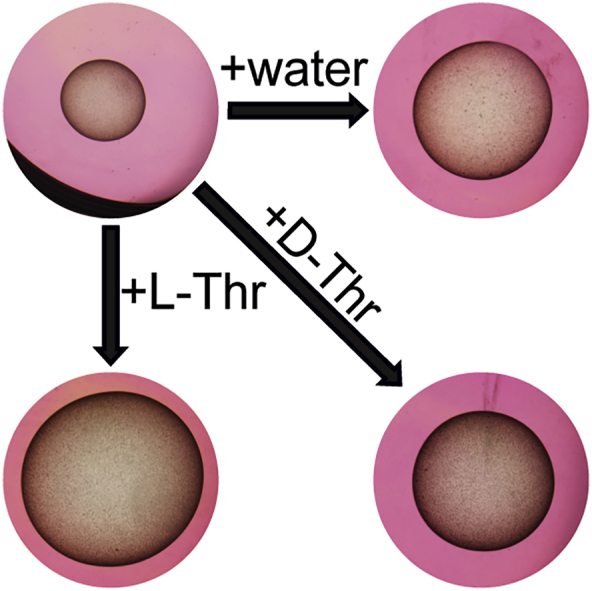

## 1 Introduction

Platyhelminth parasites pose major burdens on human and veterinary health worldwide. The class Cestoda includes the fox tapeworm *Echinococcus multilocularis* which causes the severe zoonotic disease alveolar echinococcosis (AE) in humans and other mammals such as various species of simians and dogs (Corsini et al., 2015; Eckert, 2001; Tappe et al., 2007). Worldwide, approximately 18,000 new human AE cases occur annually which correspond to 688,000 disability adjusted life years (Torgerson et al., 2015). The distribution of *E. multilocularis* is restricted to the Northern Hemisphere and more than 90% of the cases occur in China (Torgerson et al., 2010). The infection is acquired via oral uptake of *E. multilocularis* eggs, from which infective oncospheres hatch and establish themselves in the liver as metacestodes (Eckert and Deplazes, 2004). Metacestodes grow infiltratively into the liver and surrounding organs, and may form metastases to more distant body locations (Eckert and Deplazes, 2004). AE is fatal if left untreated and curative surgery is applicable in 20–50% of cases in countries with well-developed and -accessible health infrastructure (Kern et al., 2017). Nonsurgical interventions consist of lifelong therapy with daily intake of either albendazole (10–15 mg/kg/day divided in two doses) or mebendazole (40–50 mg/kg/day divided in three doses) (Brunetti et al., 2010). However, treatment with these benzimidazoles can induce adverse effects including severe liver toxicity affecting up to 6.9 % of patients (Grüner et al., 2017). The resulting treatment discontinuation can lead to recurrence of parasite growth (Grüner et al., 2017; Reuter et al., 2004), which has been proposed to be caused by the undifferentiated stem cells of metacestodes that are not affected by albendazole or mebendazole (Brehm and Koziol, 2014; Schubert et al., 2014). The undifferentiated stem cells are an integral part of the germinal layer (GL), which constitutes the actual parasite tissue and forms the inner layer of the fluid-filled metacestode vesicles (Koziol et al., 2014). In addition, the GL contains differentiated cell types such as muscle cells, nerve cells, glycogen storage cells and subtegumentary cytons (Koziol et al., 2013, 2014). The inner fluid of metacestodes, also called vesicle fluid (VF) for *in vitro* grown metacestode vesicles (Vuitton et al., 2020), stores nutrients such as glucose, as well as various amino acids (Ritler et al., 2019) and proteins (Müller et al., 2023). The GL is further surrounded by a syncytial tegument and an outer acellular and carbohydrate-rich laminated layer (Brehm and Koziol, 2017; Díaz et al., 2011). The metacestode stem cells are the only cells of the metacestode tissue that undergo continuous proliferation (Koziol et al., 2014) and, in order to be effective, new treatment options must target these stem cells (Lundström-Stadelmann et al., 2019).

In the search for new anthelmintics, efforts have been made to develop new assays for whole-organism-based drug screening that allow for efficient *in vitro* screening of drug libraries, either applying novel compounds, or repurposed drugs (Herath et al., 2022). In the case of *E. multilocularis* and the closely related *E. granulosus sensu stricto* the development of drug screening assays led to the establishment of a well-defined drug screening cascade (Kaethner et al., 2023; Lundström-Stadelmann et al., 2019). These include *in vitro* assays on the disease-causing metacestode stage and drug efficacy is assessed via damage marker and viability assays on metacestodes (Kaethner et al., 2023; Stadelmann et al., 2010, 2016), protoscoleces and isolated GL cells (Ritler et al., 2017; Stadelmann et al., 2016), which consist of up to 83% of undifferentiated stem cells (Koziol et al., 2014). While these assays are invaluable for identifying potential anti-echinococcal compounds, translating these discoveries into viable treatment options for AE remains a significant challenge. The development process involves extensive investigations on absorption, pharmacokinetics, biodistribution, toxicity, and metabolism, alongside rigorous preclinical and clinical studies. These efforts are highly resource-intensive and demand substantial financial investment. Furthermore, the market return for AE treatments is relatively low, posing additional barriers to pharmaceutical development in this field (DiMasi et al., 2016; Lundström-Stadelmann et al., 2019). This challenge can be mitigated through drug repurposing, as highlighted by Langedijk et al. (2015). Screening compounds with established pharmacological profiles against *E. multilocularis* offers a promising strategy for identifying new treatment options (Lundström-Stadelmann et al., 2020). However, the identification of novel compounds via whole-organism-based screens is costly, requires much labor and resources (Geary, 2012; Geary et al., 2015). An alternative approach involves target-based drug screening, which necessitates a deep understanding of parasite biology and the dynamics of host-parasite interaction (Geary et al., 2015).

Studies on the genome of *E. multilocularis* have revealed that the parasite has evolved to thrive within its host, relying heavily on scavenging nutrient from the host's environment to sustain its survival (Tsai et al., 2013). Examples of such adaptations are the loss of pathways for the *de novo* synthesis of fatty acids, purines and pyrimidines, cholesterol and amino acids (Coghlan et al., 2019; Tsai et al., 2013). In a previous study, we investigated the uptake of nutrients and secretion of metabolites by *E. multilocularis* metacestode vesicles in a controlled *in vitro* setting (Ritler et al., 2019). We found that metacestode vesicles take up high amounts of L-threonine (L-Thr) from the culture medium and secrete glycine (Ritler et al., 2019). Thr was not overrepresented in VF or GL cells and thus the uptake could not be explained by simple storage within these parasite compartments (Ritler et al., 2019). Albeit the laminated layer antigen Em2 is rich in Thr (Hülsmeier et al., 2002), Thr was not overrepresented in proteins of the GL or the laminated layer of *in vitro* cultured metacestode vesicles (Ritler et al., 2019). Thus, there must be another reason for the high Thr consumption of *E. multilocularis* metacestode vesicles *in vitro*.

It has been shown that in the non-parasitic model helminth *Caenorhabditis elegans*, L-Thr can be metabolized by three different enzymes: threonine deaminase (TD), threonine dehydrogenase (TDH) and threonine aldolase (TA) (Bird and Nunn, 1983; Yilmaz and Walhout, 2016) (Fig. 1). TD metabolizes L-Thr to α-ketobutyrate and ammonia (Nishimura and Greenberg, 1961). TDH catabolizes L-Thr to 2-amino-3-ketobutyrate, which is later metabolized via the 2-amino-3-ketobutyrate coenzyme A ligase (KBL) to glycine and acetyl-coenzyme A (Willetts, 1972), or can decarboxylate non-enzymatically to aminoacetone (Marcus and Dekker, 1993; Neuberger and Tait, 1960). TA-mediated L-Thr catabolism generates glycine and acetaldehyde (Lin and Greenberg, 1954). In contrast to *C. elegans*, the role and metabolization of L-Thr in parasites remains poorly understood. L-Thr catabolism via TD was investigated in the protozoan *Entamoeba histolytica* (Husain et al., 2010), the nematodes *Heligmosomoides polygyrus* and *Nippostrongylus brasiliensis* (Grantham and Barrett, 1986; Walker and Barrett, 1991) and the trematode *Fasciola indica* (Tandon and Misra, 1980). TDH-mediated L-Thr catabolism has only been characterized in the protozoan parasite *Trypanosoma brucei* (Linstead et al., 1977; Millerioux et al., 2013). Here, TbTDH has been proposed as potential drug target since human *tdh* is a nonfunctional pseudogene (Adjogatse et al., 2018; Edgar, 2002). To the best of our knowledge, no studies have been done on TA-mediated L-Thr catabolism in parasites and also studies regarding L-Thr metabolism in cestodes are lacking.

**Fig. 1:**
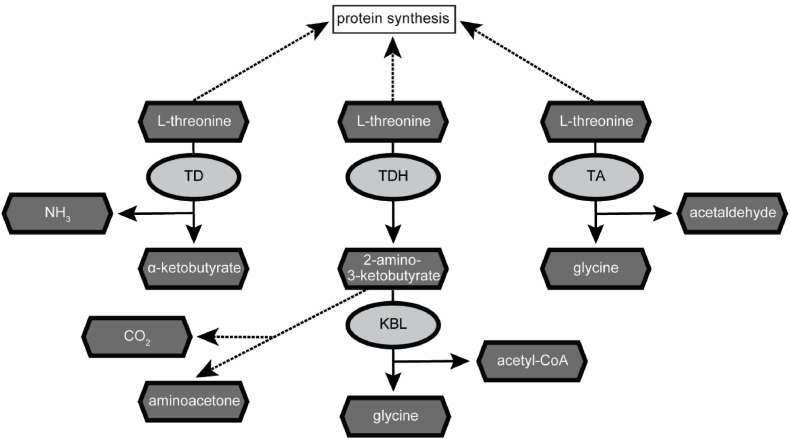
Pathways of threonine catabolism in the non-parasitic helminth *C. elegans*. L-Thr can be used as substrate by the three different enzymes threonine deaminase (TD), threonine dehydrogenase (TDH) and threonine aldolase (TA) (Yilmaz and Walhout, 2016). Upon metabolization of L-Thr by TD, α-ketobutyrate and ammonia are generated. TDH metabolizes L-Thr to 2-amino-3-ketobutyrate, which is further metabolized by the 2-amino-3-ketobutyrate coenzyme A ligase (KBL) to glycine and acetyl-coenzyme A. TA generates glycine and acetaldehyde upon degradation of L-Thr. Enzymes are depicted in light grey and metabolites in dark grey. Reactions are represented by arrows (enzymatic) or dashed arrows (non-enzymatic).

The aim of this study was to investigate the presence and relevance of an active L-Thr metabolism for *E. multilocularis in vitro*. Our findings demonstrate that an active L-Thr metabolism positively influences growth and development of *E. multilocularis in vitro*. Furthermore, we identified the enzymes involved in L-Thr catabolism and developed an enzymatic assay to evaluate potential inhibitors. These inhibitors could be further developed as drug-mediated treatment for AE in the future.

## 2 Material and methods

### 2.1 Chemicals and reagents

If not stated otherwise, all chemicals were purchased from Sigma-Aldrich (Buchs, Switzerland) and all plastic ware was purchased from Sarstedt (Sevelen, Switzerland). Dulbeccos's modified Eagle medium (DMEM) and penicillin and streptomycin (10,000 Units/mL penicillin, 10,000 μg/mL streptomycin) were from Gibco (Fisher Scientific AG, Reinach, Switzerland). DMEM without Thr and glucose was purchased from Teknova (Hollister, California, USA). Fetal bovine serum (FBS) and Trypsin/EDTA (0.05% Trypsin/0.02% EDTA) were from Bioswisstec (Schaffhausen, Switzerland). Quinazoline carboxamides (QCs) were synthesized as described in S1 File. Reuber rat hepatoma cells (RH, H-4-II-E) were purchased from ATCC (Molsheim Cedex, France).

### 2.2 Mice and ethics statement

*E. multilocularis* strain H95 was maintained in female BALB/c mice (Charles River Laboratories, Sulzheim, Germany). Mice were kept under controlled conditions with a 12 h light/dark cycle, a temperature of 21–23 °C and a relative humidity of 45–55%. Food and water were provided *ad libitum,* and cages were enriched with mouse houses (Tecniplast, Gams, Switzerland), tunnels (Zoonlab, Castrop-Rauxel, Germany) and nestlets (Plexx, Elst, Netherlands). All animals were treated in compliance with the Swiss Federal Protection of Animals Act (TSchV, SR455), and experiments were approved by the Animal Welfare Committee of the canton of Bern under the license numbers BE30/19 and BE2/22.

### 2.3 Culture of *E. multilocularis* metacestode vesicles

*E. multilocularis* metacestode vesicles (strain H95) were cultured as previously described (Kaethner et al., 2023). BALB/c mice were intraperitoneally infected with metacestode material and euthanized two to three months later. Metacestode material was aseptically collected and pressed through a conventional tea strainer with a pore size of 0.8 mm (Migros, Bern, Switzerland). The material was incubated overnight at 4 °C in PBS containing penicillin (100 U/mL), streptomycin (100 μg/mL) and tetracycline (10 μg/mL) and the next day, 1.5 mL of pure parasite material was co-cultured with RH cells in DMEM supplemented with 10% FBS, penicillin (100 U/mL), streptomycin (100 μg/mL) and tetracycline (5 μg/mL) at 37 °C under humid, 5% CO_2_ atmosphere.

### 2.4 Effect of L-Thr on *E. multilocularis*

#### 2.4.1 Development of an *E. multilocularis* metacestode vesicle growth assay

Metacestode vesicle growth was analyzed via a newly developed growth assay using automated image-based analysis in ImageJ via scripts that enables fast, precise, and objective measurements of *E. multilocularis* metacestode vesicles by providing a mean diameter of 360 radii measurements. The pre-processing of the images was performed via a cleanup algorithm adapted from a code for the measurement of tumor spheroids (Ivanov, 2015). The scripts were validated with n = 50 metacestode vesicles placed individually in 24-well plates and photographed using a Nikon SMZ18 stereo microscope (Nikon, Basel, Switzerland) at 1X magnification. The metacestode vesicles were moved within the well by circular movement of the plate to get three different images of the same metacestode vesicle. The resulting 150 images were randomly numbered and measured in a blinded manner by three different methods: a) manually in ImageJ version 1.54g with two diameters and calculations of mean values; b) by an automated macro measuring 360 radii giving mean diameter values as a result (S2 File); c) by a semi-automated version consisting of the automated script from b) and an additional user-based metacestode vesicle selection step prior to measurement (S3 File). Based on these three approaches, the scripts were validated by comparing the mean values of the metacestode vesicle diameters measured with the automated script (b) or the semi-automated script (c) to the measurements performed manually (a) in ImageJ via multiple two sample Welch tests with subsequent Bonferroni correction in R version 4.3.0 and *p*-values of *p* < 0.05 were considered to be significant. Additionally, the internal variation between the three photos of the same metacestode vesicle was calculated for all individual metacestode vesicles for each of the three measurement methods. Given are the diameter variation for the same metacestode vesicle with mean and SD values for each of the measurement methods.

#### 2.4.2 Effect of L-Thr on *E. multilocularis* metacestode vesicles

We performed a preliminary experiment to get an idea what range of L-Thr would be suitable to be tested in a growth assay with *E. multilocularis* metacestode vesicles. For this we used DMEM without Thr and glucose, added 1 mM L-Thr and conditioned it by 10^6^ RH cells for 6 day at 37 °C under a humid CO_2_ atmosphere. We sterile filtered the medium and added L-Thr (or water as control) to concentrations of 2, 4, 8 and 12 mM. Single metacestode vesicles were photographed and cultured in 1.5 mL of the different media in wells of a 24-well plate in triplicates for four days under a humid, microaerobic atmosphere (85% N_2_, 10% CO_2_, 5% O_2_). Supernatant samples were taken and stored at −20 °C for measurement of the concentration of Thr via high-performance liquid chromatography (HPLC) at the Department of Chemistry, Biochemistry and Pharmaceutical Sciences, University of Bern (see S4 File). Metacestode vesicle size was measured via the semi-automated script (2.4.1.c, S3 File). The respective reduction of L-Thr in the culture medium was measured by HPLC and significance was assessed via multiple two sample Welch tests and subsequent Bonferroni correction in R. Bonferroni-correct *p*-values of *p* < 0.05 were considered to be significant. Shown are the metacestode vesicle diameter with mean values and SDs, as well as the reduction of L-Thr in the culture medium with mean values and SDs.

For the metacestode growth assay, metacestode vesicles cultured for three to four months with a mean diameter of 3.3 mm ± 0.5 mm were changed to an axenic culture system without RH cells as described by others (Spiliotis and Brehm, 2009). A6 medium was prepared from low glucose DMEM (1 g/L glucose) supplemented with 10% FBS, penicillin (100 U/mL), streptomycin (100 μg/mL) and tetracycline (5 μg/mL) by conditioning with 10^6^ RH cells for 6 day at 37 °C under a humid CO_2_ atmosphere and subsequent sterile filtration. The medium was stored at 4 °C not longer than one week. n = 24 single metacestode vesicles per condition were distributed individually in 24-well plates and incubated in 1.5 mL A6 medium. In a first experiment, L-Thr was added to final concentrations of 1, 2, or 4 mM. Alternatively, D-Thr was added to 4 mM final concentration. These concentrations were chosen based on the data obtained from the preliminary experiment (see above), with the goal of observing a potential dose-dependent effect. An equal amount of distilled water was added to the control. In an independent second experiment, we also wanted to assess the effect of the non-proteinogenic Thr analogue 3-hydroxynorvaline (3-HNV) (Kazuoka et al., 2003; Wang et al., 2009). Thus, we performed an assay in which we supplemented 4 mM 3-HNV, a combination of 4 mM 3-HNV and 4 mM L-Thr, or the respective amount of distilled water to the media of individually placed metacestode vesicles. Both experiments were performed two times independently with 24 replica per condition, metacestode vesicles were incubated under microaerobic conditions and medium changes were performed once a week.

For assessment of parasite growth, metacestode vesicles were photographed at the start and the end of the experiment using a Nikon SMZ18 stereo microscope at 0.75X magnification. A lower magnification was chosen than in 2.4.1 due to the expected increase of metacestode vesicle diameters after six weeks of incubation. Metacestode vesicle diameters were assessed via the automated script (2.4.1.b, S2 File) and in case the macro did not work perfectly (due to metacestode vesicles being too close to the border of the well), images were processed with the modified, semi-automated version of the script (2.4.1.c, S3 File) in which the metacestode vesicle is manually encircled.

At weeks 0 and 6, all groups were compared to either the control (first experiment), or to 4 mM 3-HNV (second experiment) via multiple two sample Welch tests with subsequent Bonferroni correction in R. Bonferroni-corrected *p*-values of *p* < 0.05 were considered to be significant.

#### 2.4.3 Isolation of GL cells from *E. multilocularis* metacestode vesicles

GL cells were isolated as described by a recently updated protocol (Kaethner et al., 2023). In short, conditioned DMEM (cDMEM) was prepared by culturing high-glucose DMEM supplemented with 10% FBS, penicillin (100 U/mL), streptomycin (100 μg/mL) and tetracycline (5 μg/mL) with 10^6^ Rh cells in 50 mL medium for six days, and 10^7^ cells in 50 mL medium for 4 days at 37 °C under a humid, 5% CO_2_ atmosphere and after sterile filtration, combining them 1:1. Six-months-old metacestode vesicles were incubated in distilled water for 2 min, washed with PBS and mechanically broken using a pipette. The vesicle tissue (VT) was washed in PBS and incubated in eight volumes trypsin-EDTA solution at 37 °C for 30 min. GL cells were extracted by filtering through a 30 μm mesh (Sefar AG, Heiden, Switzerland) and separated from calcareous corpuscles by short centrifugation (50×*g*, 30 s). The cells were centrifuged, re-suspended in cDMEM and a 1:100 dilution was used to measure the OD_600_. An OD_600_ value of 0.1 of this dilution was defined as one arbitrary unit (AU) per μL of the undiluted cell suspension. 1 AU corresponded to 0.93 ± 0.17 μg total protein for eight different GL cell isolations of this study, as determined by bicinchoninic acid (BCA) assay using the Pierce™ BCA Protein Assay Kit (Fisher Scientific AG, Reinach, Switzerland). 1000 AU of GL cells were cultured in five mL cDMEM at 37 °C overnight under a humid nitrogen atmosphere. The next day, 2000 AU of GL cells were combined and further cultured for 3 h at 37 °C under a humid nitrogen atmosphere.

#### 2.4.4 Vesicle formation assay

Vesicle formation assays were carried out as described by Hemer and Brehm (2012) with a few modifications such as a microaerobic atmosphere and a less enriched medium. In short, 150 AU of *E. multilocularis* GL cells were cultured in a 96-well plate in high glucose DMEM containing 1% FBS and penicillin (100 U/mL), streptomycin (100 μg/mL) and tetracycline (5 μg/mL) under a humid, microaerobic atmosphere. In a first experiment, 4 mM L-Thr, 4 mM D-Thr, or the respective amount of distilled water was added to the GL cells. In an independent second experiment, 4 mM 3-HNV, a combination of 4 mM L-Thr and 4 mM 3-HNV, or the respective amount of distilled water was added. Both experiments were setup in four technical replica and this experiment was performed twice (independent biological replicates). Three times a week, half of the medium in each well was changed. After two weeks, newly formed metacestode vesicles were counted in a blinded manner. Shapiro-Wilk tests showed normal distribution with *p* > 0.05 for all groups of each experiment. Statistical analyses were performed using multiple two-tailed students t-tests with equal variance. The Bonferroni-corrected *p*-values of *p* < 0.05 were considered to be significant.

### 2.5 Tracing [U-^13^C] L-Thr and metabolites in *E. multilocularis* metacestode vesicles

We studied how L-Thr is metabolized in *E. multilocularis in vitro* by tracing [U-^13^C]-L-Thr and metabolites in metacestode vesicles and culture medium. 3 mL of two- to three-months-old *E. multilocularis* metacestode vesicles were cultured in 3 mL DMEM without Thr or glucose, supplemented with 0.2% FBS, 110 mg/L sodium pyruvate, 4.5 g/L glucose, 4 mM L-glutamine and 5 mM unlabeled Thr or 5 mM [U-^13^C]-L-Thr, respectively, at 37 °C for 24 h under a humid, microaerobic atmosphere. Control medium (CM) without metacestode vesicles was incubated under the same conditions. Each condition was set up in four biological replicates. Medium samples of CM and assay medium in which metacestode vesicles were incubated (VM), metacestode VF and metacestode VT were extracted in an ice-cold buffer consisting of HPLC grade chloroform:methanol:water (1:3:1 ratio). Medium samples were centrifuged (13,000×*g*, 5 min, 4 °C), 10 μL of supernatant were mixed with 400 μL of extraction buffer by vortexing and centrifuged again (13,000×*g*, 5 min, 4 °C). The supernatant was stored at −80 °C. Metacestode vesicles were washed three times in 50 mL ice-cold PBS and then mechanically disrupted with a pipette. The VF was centrifuged (13,000×*g*, 5 min, 4 °C) and 10 μL of supernatant were mixed with 400 μL of extraction buffer by vortexing. The sample was centrifuged again (13,000×*g*, 5 min, 4 °C) and the supernatant was stored at −80 °C. The metacestode VT pellet was washed three times with 1 mL ice-cold PBS and a centrifugation step (500×*g*, 5 min, 4 °C). The pellet was homogenized in 10 mL extraction buffer by vortexing with a 5 mm glass bead (30 steps of vortexing (10 s) and resting on ice (50 s)). The sample was centrifuged (4700×*g*, 5 min, 4 °C), the supernatant was centrifuged again (13,000×*g*, 5 min, 4 °C) and the supernatant was stored at −80 °C. Blanks consisted of extraction buffer using the same tubes and vortexing/centrifugation steps. For tracing of [U-^13^C]-L-Thr and metabolites, samples were analyzed via Hydrophilic interaction liquid chromatography (HILIC) on a Dionex UltiMate 3000 RSLC system (Thermo Fisher Scientific, Hemel Hempstead, UK) using a ZIC-pHILIC column (150 mm × 4.6 mm, 5 μm column, Merck Sequant). The column was maintained at 25 °C and samples were eluted over 26 min at a flow rate of 0.3 mL/min with a linear gradient over 15 min from an initial ratio of 80% acetonitrile (B) and 20% 20 mM ammonium carbonate in water (A) to 20% B and 80% A, followed by 95% A and 5% B for 2 min followed by re-equilibration at 80% B and 20% A for 9 min. The injection volume was 10 μL and samples were maintained at 5 °C prior to injection. For the MS analysis, a Thermo Orbitrap QExactive (Thermo Fisher Scientific) was operated in polarity switching mode and the MS settings were Resolution 70,000, AGC 1e6, *m*/z range 70–1050, sheath gas 40, auxiliary gas 5, sweep gas 1, probe temperature 150 °C and capillary temperature 320 °C. The samples were analyzed in positive mode ionization (source voltage +3.8 kV, S-Lens RF Level 30.00, SLens Voltage 25.00 V, Skimmer Voltage 15.00 V, Inject Flatopole Offset 8.00 V, Bent Flatapole DC 6.00 V) and negative mode ionization (source voltage −3.8 kV). The calibration mass range was extended to cover small metabolites by inclusion of low-mass calibrants with the standard Thermo calmix masses (below *m/z* 138), butylamine (C_4_H_11_N_1_) for positive ion electrospray ionization (PIESI) mode (*m/z* 74.096426) and COF3 for negative ion electrospray ionization (NIESI) mode (*m/z* 84.9906726). For each sample subset (medium, VF and VT), LC-MS raw data was processed with IDEOM (Creek et al., 2012) which uses the XCMS (Smith et al., 2006) and mzMatch software (Scheltema et al., 2011) in the R environment. A list of putatively annotated metabolites was generated and the abundances of all [^13^C]- isotopologues for these were obtained using the software mzMatch-ISO (Chokkathukalam et al., 2013). Metabolomics data have been deposited to the EMBL-EBI MetaboLights database (https://doi.org/10.1093/nar/gkad1045, PMID:37971328) with the identifier MTBLS10738. For relevant metabolites the area under the curve (AUC) is shown as mean and SD values in S3 Table. In addition, we calculated the relative fractional enrichment in % for each [^13^C]- isotopologue of relevant metabolites by dividing its abundance through the sum of abundances of the other [^13^C]- isotopologues of this respective metabolite. The mean and SD values are also given in S3 Table.

### 2.6 Expression and activity of Thr metabolism genes in *E. multilocularis* metacestode vesicles *in vitro*

#### 2.6.1 Genes of Thr metabolism in *E. multilocularis*

The following protein sequences of Thr metabolism were blasted against the protein database of *E. multilocularis* PRJEB122 (Tsai et al., 2013) via WormBase ParaSite (https://parasite.wormbase.org, assessed on 10/17/2023): TD, TDH, KBL and TA from reference organisms *Caenorhabditis elegans, Danio rerio, Drosophila melanogaster*, *Homo sapiens* and *Mus musculus* (if present as functional proteins, see S1 Table). These sequences were obtained from UniProt (https://www.uniprot.org, assessed on 10/17/2023). Human *ta* and *tdh* were excluded since they are pseudogenes (Edgar, 2002, 2005) and *td* is not present in *D. rerio*. Top hits within the protein database of *E. multilocularis* were then blasted reciprocally against the NCBI non-redundant protein database of the reference organisms (https://blast.ncbi.nlm.nih.gov/Blast.cgi).

We performed the same approach to identify TDH sequences in the closely related parasites *E. granulosus s.s.* with the protein database PRJEB121. We then aligned TDH amino acid sequences of the reference organisms *C*. *elegans* (Q22945)*, D*. *rerio* (Q6P3J8)*, D*. *melanogaster* (Q9VPE8), *H*. *sapiens* (Q8IZJ6) and *M*. *musculus* (Q8K3F7) with TDH sequences of *E. multilocularis* (EmuJ_000511900) and *E. granulosus s.s.* (EgrG_000511900). The alignment was generated with BioEdit 7.2 (Hall, 1999).

#### 2.6.2 Preparation of *E. multilocularis* metacestode vesicles and *in vivo* grown metacestodes for assessment of Thr metabolism gene expression

In order to study whether Thr metabolism of *in vitro* cultured metacestode vesicles of isolate H95 under axenic, microaerobic culture conditions was similar to previously published data from isolate G8065 (Tsai et al., 2013), we analyzed gene expression via quantitative real-time PCR for the four genes, *emtd* (EmuJ_001093200), *emtdh* (EmuJ_000511900), *emkbl* (EmuJ_000107200) and the house-keeping gene *ezrin/radixin/moesin-like protein* (*emelp*) (EmuJ_000485800) (Brehm et al., 2003; del Puerto et al., 2016). Five months old *E. multilocularis* metacestode vesicles were purified and changed to an axenic culture system without RH cells as described by others (Spiliotis and Brehm, 2009) and incubated under humid microaerobic atmosphere for two days. Metacestode vesicles were mixed with three volumes of A6 medium (see 2.4.2 but using DMEM with 4.5 g/L of glucose), and 4 mL were distributed to 6-well plates. Each condition was set up in four biological replica and two independent experiments were conducted. Metacestode vesicles were incubated under humid, microaerobic atmosphere for three days. Metacestode vesicles were washed three times in PBS, destroyed with a pipette and again washed three times in PBS with a centrifugation step (600×*g*, 3 min, 4 °C) after each washing step. The metacestode VT was taken up in 1.8 mL TRI Reagent®, shaken at 1400 RPM in an Eppendorf® Thermomixer Compact (Vaudaux Eppendorf, Schönenbuch, Switzerland) for 15 min at RT and frozen to −20 °C until RNA extraction.

BALB/c mice were intraperitoneally injected with *E. multilocularis* metacestode tissue for routine strain maintenance (see also 2.2). Metacestode tissue from four individual mice was washed in PBS, mechanically disrupted and homogenized in 1.8 mL TRI Reagent® in a 2 mL screw cap tube with a five mm glass bead in a FastPrep-24TM Classic homogenizer (MP Biomedicals, Illkirch-Graffenstaden, France) with five cycles of 4 m/s for 20 s. Then, samples were shaken at 1400 RPM in an Eppendorf® Thermomixer Compact for 15 min at RT and centrifuged at (12,000×*g*, 10 min, 4 °C). The supernatant was transferred to a new tube and samples were frozen to −20 °C until RNA extraction.

#### 2.6.3 Isolation of RNA from *E. multilocularis* metacestode vesicles and metacestode cysts

RNA from *E. multilocularis* metacestode vesicles and metacestode cyst tissue was isolated via a phenol/chloroform extraction as described by others (Pérez et al., 2019) with a few modifications. In short, 0.2 vol of chloroform were added per mL TRI Reagent® to each sample, they were incubated at RT for 3 min and subsequently centrifuged (12,000×*g*, 15 min, 4 °C). The aqueous phase was taken, and samples were pipetted on parafilm to remove residual chloroform, mixed with 1 mL isopropanol and incubated at RT for 10 min. After centrifugation (12,000×*g*, 15 min, 4 °C) the pellets were washed once in 75% ethanol and centrifuged (7500×*g*, 5 min, 4 °C). The pellets were air-dried, and DNA digestion was performed with the Direct-zol RNA Miniprep Kit from Zymo Research (Lucerna-Chem AG, Lucerne, Switzerland). The RNA was resuspended in 87.5 μL RNAse-free water, mixed with 20 μL DNA Digestion Buffer and 5 μL DNAse I. Samples were incubated at RT for 10 min and then 0.1 vol of 3 M Diethyl pyrocarbonate-treated sodium acetate, pH 5.2, and 2.5 vol of 100% ethanol were added. Samples were incubated at −80 °C for 1.5 h. The samples were centrifuged (16,000×*g*, 10 min, 4 °C) and the pellet washed once in 75% ethanol and centrifuged (7500×*g*, 5 min, 4 °C). Pellets were air-dried, resuspended in RNAse-free water and concentrations were measured via a NanoDrop™ One/OneC Microvolume UV–Vis Spectrophotometer (Thermo Fisher Scientific, Reinach, Switzerland). 1 μg of RNA was reverse transcribed via the GoScript™ Reverse Transcription System (Promega, Dübendorf, Switzerland) in a final volume of 20 μL.

#### 2.6.4 Quantitative real-time PCR of *E. multilocularis* RNA samples

Gene expression was analyzed via specific, intron-flanking primers, which are shown in in S2 Table. Quantitative real-time PCRs were performed on a CFX Opus 96 Real-Time PCR System (Biorad). Primer efficiency was calculated by performing RT-PCR reactions using 1 μL of cDNA, and 1 μL of four subsequent 1:4 dilutions as template, except for *emtd* for which 1:2 dilutions were made, due to the expected lower expression (Tsai et al., 2013). Gene expression was analyzed in technical duplicates for each biological quadruplicate and calculated relative to *emelp*. Two independent experiments were performed using RNA from *in vitro* cultured metacestode vesicles and one experiment was performed using RNA from *ex vivo* metacestodes obtained from experimentally infected mice. Ct values corresponding to all analyzed genes were normalized to *emelp* and data is shown as relative fold-change with mean values and SD.

#### 2.6.5 Enzymatic activity of EmTDH in crude extracts of *E. multilocularis* metacestode vesicles

For the preparation of crude extracts of *E. multilocularis* metacestode vesicles, parasite vesicles grown for at least 6 months *in vitro* were ruptured by pipette and the pellet was washed three times in PBS. The pellet was taken up in a lysis buffer (80 mM Tris-HCl pH 8.4 supplemented with 1% Triton X-100, 1% Halt™ Protease Inhibitor Cocktail (Thermo Fisher Scientific) and 1% EDTA) in a volume where 1 mL of buffer corresponded to 10 mL pure, intact metacestode vesicles. The protein amount was determined by the Pierce™ BCA Protein Assay Kit and resulted in 8 mg/mL.

We established an assay for the characterization of enzymatic activity of EmTDH within crude extract of *E. multilocularis* metacestode vesicles. The TDH assay was adapted for *E. multilocularis* metacestode vesicles using a protocol for *E. coli* TDH as a basis (Craig and Dekker, 1986). The enzymatic assay buffer consisted of 80 mM Tris HCl (pH 8.4) and 10 mM NAD. We incubated 80 μg protein crude extract of *E. multilocularis* metacestode vesicles per well with various concentrations of L-Thr and D-Thr (0, 1, 2, 4, 8, 16 mM) in technical triplicates. Enzyme blanks were included. The reaction was measured at 37 °C via an increase in absorbance at 340 nm on a HIDEX Sense microplate reader (Hidex, Turku, Finland). Enzyme blanks were subtracted from the results and shown are mean values and SDs.

### 2.7 Inhibition of *E. multilocularis* Thr metabolism by TDH inhibitors

#### 2.7.1 TDH assay with recombinantly expressed EmTDH and MmTDH

The sequence of *emtdh* was obtained from WormBase ParaSite (https://parasite.wormbase.org) via the accession number EmuJ_000511900 and the sequence of *mmtdh* was obtained from the National Library of Medicine (https://www.ncbi.nlm.nih.gov) via the accession number ENSMUST00000022522.15. Both sequences, as well as the detailed cloning process with images is shown in S5 File.

Briefly, *emtdh* (EmuJ_000511900) was amplified from cDNA of *in vitro* grown *E. multilocularis* metacestode vesicles without the predicted mitochondrial target sequence as a 979 bp sequence. The amplification of *mmtdh* (ENSMUST00000022522.15) was not possible, due to low expression in tissue of adult mice (Wang et al., 2009) and was therefore ordered as a 1007 bp fragment from LubioScience (Lucerne, Switzerland) without the predicted mitochondrial transfer peptide sequence, and amplified with a resulting size of 991 bp. Both *emtdh* and *mmtdh* were cloned into the pET151/D-TOPO® vector using the Champion™ pET151 Directional TOPO™ Expression Kit (Fisher Scientific AG, Reinach, Switzerland). Clones were picked and tested via colony PCR for the expected fragment size and correct orientation with a vector-specific forward primer and an insert-specific reverse primer. Plasmid DNA was isolated from positive clones using the ZymoPURE™ Plasmid Miniprep Kit (Zymo Research, Irvine, USA) and Sanger sequencing was conducted at Microsynth AG (Balgach, Switzerland). Sequences were compared in BioEdit (Hall, 1999) to their respective reference sequence and correct plasmids were used to transform *E. coli* BL21 for recombinant expression of His-tagged EmTDH as a 365 amino acids protein and His-tagged MmTDH as a 361 amino acid protein. RecEmTDH and recMmTDH were purified via the Macherey-Nagel™ Protino™ Ni-TED-IDA 1000 Kit (Fisher Scientific, Schwerte, Germany) according to the manufacturer's protocol and eluates were checked on a 12% sodium dodecyl sulfate polyacrylamide gel. Correct protein size of 41.4 kDa for recEmTDH and 40.3 kDa for recMmTDH was confirmed by Western blot using a mouse monoclonal anti-His tag antibody and an anti-mouse IgG (h + l) ap conjugate as secondary antibody (Promega, Dübendorf, Switzerland). Finally, protein concentration of the eluates was determined via BCA assay using the Pierce™ BCA Protein Assay Kit.

#### 2.7.2 Inhibition of recEmTDH and recMmTDH

We first tested the activity of recEmTDH via the established TDH assay (see 2.6.6) using 0.05 μg of recEmTDH per well with 10 mM NAD^+^ and a variety of amino acids (3-HNV, D-Thr, glycine, L-alanine, L-cysteine, L-serine and L-Thr) at a concentration of 16 mM. We also tested whether the enzyme can use NADP^+^ instead of NAD+ as a cofactor by measuring recEmTDH activity with 4 mM L-Thr and 10 mM NADP+. We then performed kinetic studies on recEmTDH by evaluating TDH activity with 0.05 μg of recEmTDH per well with concentration series of either NAD^+^ (0, 0.125, 0.25, 0.5, 1, 2, 4 and 8 mM) and 16 mM L-Thr, or concentration series of L-Thr (0, 0.25, 0.5, 1, 2, 4, 8 and 16 mM) and 2 mM NAD^+^. Kinetic parameters (V_max_ and K_m_) for the respective substrate were calculated using non-linear least square fitting of the Michaelis-Menten equation. We then tested recMmTDH using 0.05 μg of recMmTDH per well with 10 mM NAD^+^ and a concentration series of L-Thr or D-Thr (0, 1, 2, 4, 8 and 16 mM). For all experiments, each condition was performed in technical triplicates and substrate blanks (without recEmTDH or recMmTDH) and enzyme blanks (without addition of L-Thr) were included. Enzymatic activity was measured at 37 °C via an increase in absorbance at 340 nm on a HIDEX Sense microplate reader. Enzyme blanks were subtracted from the enzymatic reaction wells.

For the subsequent inhibition experiments, the assay buffer was supplemented with 2 mM L-Thr, 3 mM NAD^+^ and 0.05 μg recEmTDH, or 0.05 μg recMmTDH, per well of a 96-well plate. Several published inhibitors of Thr metabolism were tested on recEmTDH and recMmTDH in the established TDH assay, namely disulfiram, myricetin, quercetin, seven quinazoline carboxamides (QC) and sanguinarine (Adjogatse, 2015; Alexander et al., 2011; Cross et al., 1975). The inhibitors were added at 20 μM in technical triplicates and enzyme activity was normalized to respective DMSO controls. Significant inhibition compared to the DMSO control was assessed via multiple two-sample one-tailed students t-tests assuming equal variance and subsequent Bonferroni-correction in R. Inhibitors were considered active when reduction of enzyme activity was significant according to Bonferroni-corrected *p*-values of *p* < 0.05.

### 2.8 Assessment of moderate EmTDH inhibitors on *E. multilocularis in vitro*

#### 2.8.1 Phosphoglucose isomerase (PGI) assay on *E. multilocularis* metacestode vesicles

Damage marker release assays, based on the marker PGI, were performed as described previously (Stadelmann et al., 2010) with the modifications published recently (Kaethner et al., 2023). In short, two-to three-months-old metacestode vesicles were purified with 2% sucrose and several washing steps in PBS. Metacestode vesicles were mixed with two volumes of high glucose DMEM without phenol red containing penicillin (100 U/mL) and streptomycin (100 μg/mL). 1 mL of the metacestode vesicle-medium mix was distributed into a 48-well plate (Huberlab, Aesch, Switzerland), and the three most potent, though neither specific nor highly active, recEmTDH inhibitors - disulfiram, myricetin and sanguinarine - were added to final concentrations of 20 μM in triplicates. The respective amount of DMSO was used as a negative control and 0.1% Triton X-100 was used as a positive control. The plate was incubated at 37 °C under a humid, microaerobic atmosphere and pictures and supernatant samples were taken after five days. Supernatant samples were measured on a HIDEX Sense microplate reader. The corresponding values of the DMSO controls were subtracted from the values of the compounds and then PGI activity was calculated relative to 0.1% Triton X-100. Given are mean values and SD. Significant differences of compound-treated metacestode vesicles compared to the DMSO control were calculated via two-sample two-tailed students t-test with equal variance and subsequent Bonferroni-correction in R. Bonferroni-corrected *p*-values of *p* < 0.05 were considered to be significant.

#### 2.8.2 GL cell viability assay

Cell viability assays with *E. multilocularis* GL cells were carried out as described recently (Kaethner et al., 2023). In short, 15 AU of GL cells (see 2.4.3 for extraction of GL cells) were distributed into wells of a black 384-well plate in a volume of 12.5 μL. In another 12.5 μL, disulfiram, myricetin and sanguinarine were added to final concentrations of 20 μM, respective amounts of DMSO and Triton X-100 (0.1%) were included as controls. For overview screening, each drug was tested in quadruplicates. Cells were incubated at 37 °C under a humid, microaerobic atmosphere for five days. Pictures were taken and to each well, 25 μL of CellTiter-Glo containing 1% Triton X-100 was added and cell aggregates were disrupted by pipetting. Luminescence was recorded on a HIDEX Sense microplate reader and mean values and SD were calculated. Significant differences of compound-treated GL cell cultures compared to the DMSO control were calculated via two-sample two-tailed students t-test with equal variance and subsequent Bonferroni-correction in R. Bonferroni-corrected *p*-values of *p* < 0.05 were considered to be significant. Two independent experiments were performed for the overview screen.

## 3 Results

### 3.1 Thr metabolism promotes *E. multilocularis* metacestode vesicle growth and development *in vitro*

To study the effect of Thr on *E. multilocularis* growth *in vitro*, we developed a metacestode vesicle growth assay using an automated and a semi-automated script in ImageJ to precisely and objectively follow the growth of single metacestode vesicles over time. The scripts were validated with 150 photos of 50 individual metacestode vesicles being photographed each three times. The mean vesicle diameter and SDs are shown in S1 Fig. There was no significant difference between measurement of metacestode vesicle diameter via the automated script, the semi-automated script, or the manual measurement performed in ImageJ. The mean internal diameter variance between the three images of each metacestode vesicle was 1.3% for the manual measurements and 1.2% for both the automated and the semi-automated scripts. Due to the general low variance between the photos, the images of the metacestode vesicle growth assays were analyzed with the automated script and in case vesicles were not accurately detected, images were analyzed with the semi-automated script.

In a preliminary experiment the most stuitable L-Thr concentration for a growth assay was assessed. Under the described culture conditions, addition of 4 mM of L-Thr led to the highest reduction of L-Thr in the culture medium (S2 Fig). Thus, 4 mM L-Thr was used as a maximal concentration to be tested subsequently.

For experiment one, *E. multilocularis* metacestode vesicles were cultured *in vitro* in medium supplemented with L-Thr at 1, 2 and 4 mM, or 4 mM D-Thr, (Fig. 2A). Of a total of 384 metacestode vesicles analyzed, seven (1.8%) collapsed within the six weeks of the experiment and thus these 14 images from week 0 and week 6 were excluded from the analysis. Of the remaining 754 images, 696 images (92.3%) were detected correctly by the automated script. The 55 images (7.3%) in which the metacestode vesicles were not accurately detected were processed with the semi-automated script. Over the time course of six weeks, metacestode vesicles supplemented with water as a control reached a diameter of 5.5 mm ± 0.6 mm. The metacestode vesicle diameter was significantly increased to 6.0 mm ± 0.5 mm when metacestode vesicles were cultured with 1 mM L-Thr (*p* = 0.012), to 6.2 mm ± 0.9 mm (*p* = 0.01) with 2 mM L-Thr and to 6.4 mm ± 1.0 mm (*p* = 0.003) with 4 mM L-Thr. Supplementation with 4 mM D-Thr led to a metacestode vesicle diameter of 5.4 mm ± 0.7 mm, which was not significantly different from the control. Thus, L-Thr but not D-Thr led to a significant stimulation of *E. multilocularis* metacestode vesicle growth *in vitro.* The experiment was repeated once independently, and we obtained similar results with a significant increase of metacestode vesicle growth with increasing concentrations of L-Thr but not D-Thr (S3 Fig).

**Fig. 2:**
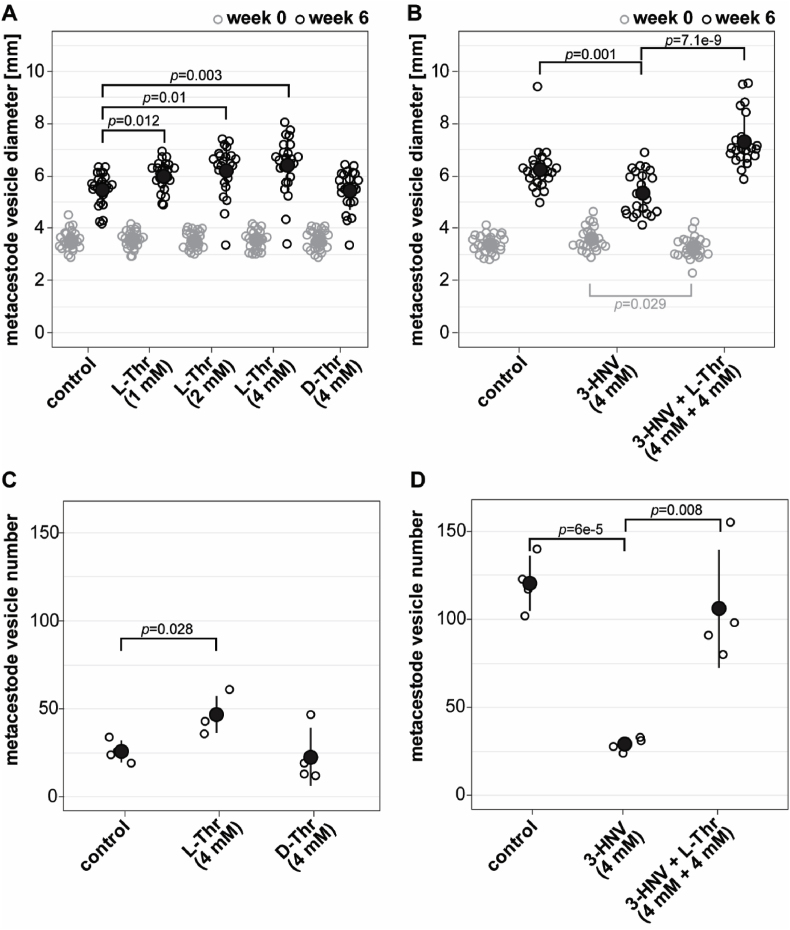
L-Thr stimulates *E. multilocularis* metacestode vesicle growth and development *in vitro*. A, *E. multilocularis* metacestode vesicles were cultured with various concentrations of L-Thr (1 mM, n = 24; 2 mM, n = 23; 4 mM, n = 23), D-Thr (4 mM, n = 23), or water as a control (n = 24). B, *E. multilocularis* metacestode vesicles were cultured in the presence of 3-HNV (4 mM, n = 21), a combination of 3-HNV and L-Thr (each at 4 mM, n = 23), or water (n = 24). Metacestode vesicle diameters in A and B were measured via an automated script and are given in mm at weeks 0 (grey) and 6 (black). Bonferroni-corrected *p-*values are displayed as compared to the water control or 4 mM 3-HNV. C, *E. multilocularis* GL cell cultures were supplemented with L-Thr (4 mM, n = 4), D-Thr (4 mM, n = 4), or water as a control (n = 4). D, *E. multilocularis* GL cells were cultured with 3-HNV (4 mM, n = 4), a combination of 3-HNV and L-Thr (both at 4 mM, n = 4), or water as a control (n = 4). Newly formed metacestode vesicles in C and D were counted manually in a blinded manner and the mean values, SD and Bonferroni-corrected *p-*values are shown. Abbreviations: Thr = threonine, 3-HNV = 3-hydroxynorvaline. Two independent experiments were performed for each setup, and the results of one representative experiment is shown, while the results of the second experiment are provided in S3 Fig.

In a second experiment (Fig. 2B), *E. multilocularis* metacestode vesicles reached a diameter of 6.2 mm ± 0.8 mm within the six weeks of this experiment and this growth was significantly reduced upon incubation with 4 mM 3-HNV (5.3 mm ± 0.8 mm, *p* = 0.001). The combined incubation of 4 mM 3-HNV and 4 mM L-Thr compensated for this reduction significantly (7.3 mm ± 1 mm, *p* = 7.1e-9). Upon independent repetition of the experiment, we obtained similar results with significant reduction in growth for metacestode vesicles treated with 4 mM 3-HNV. This effect was significantly counteracted upon treatment with a combination of 4 mM 3-HNV and 4 mM L-Thr (S3 Fig).

We then assessed the effects of Thr on the formation of new metacestode vesicles from GL cells. In the control culture supplemented with water 26 ± 5 metacestode vesicles were formed within two weeks (Fig. 2C). This formation was significantly increased upon addition of 4 mM L-Thr (47 ± 9 metacestode vesicles, *p* = 0.028), but not with the addition of D-Thr (23 ± 14 metacestode vesicles). We repeated this experiment once independently and obtained a similar trend, but not a significant difference between the control and L-Thr (see S3 Fig).

We assessed the effect of 3-HNV with and without L-Thr accordingly on the metacestode vesicle formation rate (Fig. 2D). While supplementation of water resulted in the formation 121 ± 14 metacestode vesicles from GL cell cultures, we observed a significant reduction of vesicle formation when incubated with 4 mM 3-HNV (29 ± 3 metacestode vesicles, *p* = 6e-5). As observed in the metacestode vesicle growth assay, the combined supplementation of 4 mM 3-HNV and 4 mM L-Thr significantly counteracted this reduction (106 ± 26 metacestode vesicles, *p* = 0.008). This experiment was repeated once independently, and we obtained similar and significant results (S3 Fig).

### *3.2* *E. multilocularis* metacestode vesicles metabolize L-Thr to glycine via EmTDH

In order to identify relevant pathways through which L-Thr is metabolized by *E. multilocularis*, we performed a flux experiment with [U-^13^C]-L-Thr and detected labeled metabolites by LC-MS in different fractions of metacestodes (VT, VF, CM and VM). Labeling patterns are visualized in Fig. 3 and for the detected metabolites relevant to L-Thr metabolism (L-Thr, glycine and aminoacetone) individual values of fractional enrichment with raw AUC and relative values are shown in S3 Table. We detected [U-^13^C]-L-Thr in VT, VF, CM and VM (93 ± 0.4%, 94.2 ± 0.1%, 96.1 ± 0% and 95.8 ± 0.1%, respectively) indicating an uptake of L-Thr by *E. multilocularis* metacestode vesicles and transportation to the VF. The direct metabolic product of TD-mediated L-Thr catabolism, α-ketobutyrate, was not detected in any of the samples. The direct product of TDH-mediated L-Thr catabolism, 2-amino-3-ketobutyrate, an unstable product, was not detected either. However, we detected [U-^13^C]-aminoacetone in VM (64.6 ± 5.6%), which is generated upon spontaneous decarboxylation of 2-amino-3-ketobutyrate (Marcus and Dekker, 1993). We further detected [U-^13^C]-glycine in VT, VF and VM (42.7 ± 2.4%, 30.1 ± 2.3% and 28 ± 3.3%, respectively), but only minor traces in CM (0.2 ± 0.2%) indicating that L-Thr metabolism fed into glycine production. Besides glycine, acetyl-coenzyme A is also generated via KBL-mediated metabolization of 2-amino-3-ketobutyrate. We detected various TCA cycle intermediates, namely citrate, its downstream metabolite α-ketoglutarate and its derivative 2-hydroxyglutarate, succinate, malate, and the transamination product of oxaloacetate, L-aspartate. None of these metabolites were found to be [^13^C]-labeled. Thus, our results do not suggest the use of L-Thr metabolites within the TCA cycle in *E. multilocularis* metacestode vesicles. Further, we detected [^13^C_2_]-glutathione in VT samples (6.6 ± 0.5%), which indicates that L-Thr-derived glycine fed into the biosynthesis of glutathione. Additionally, we found [^13^C_2_]-L-glutamate in VT with 2 ± 0.2% and some traces in VF and VM (0.3 ± 0.3% and 0.1 ± 0.1%, respectively), while CM was completely unlabeled, which indicates a L-Thr-derived formation of L-glutamate by *E. multilocularis in vitro*. We detected palmitate and propionate in our samples, but also in the blank samples and neither of these metabolites contained [^13^C]. Thus, the data was not exploited.

**Fig. 3:**
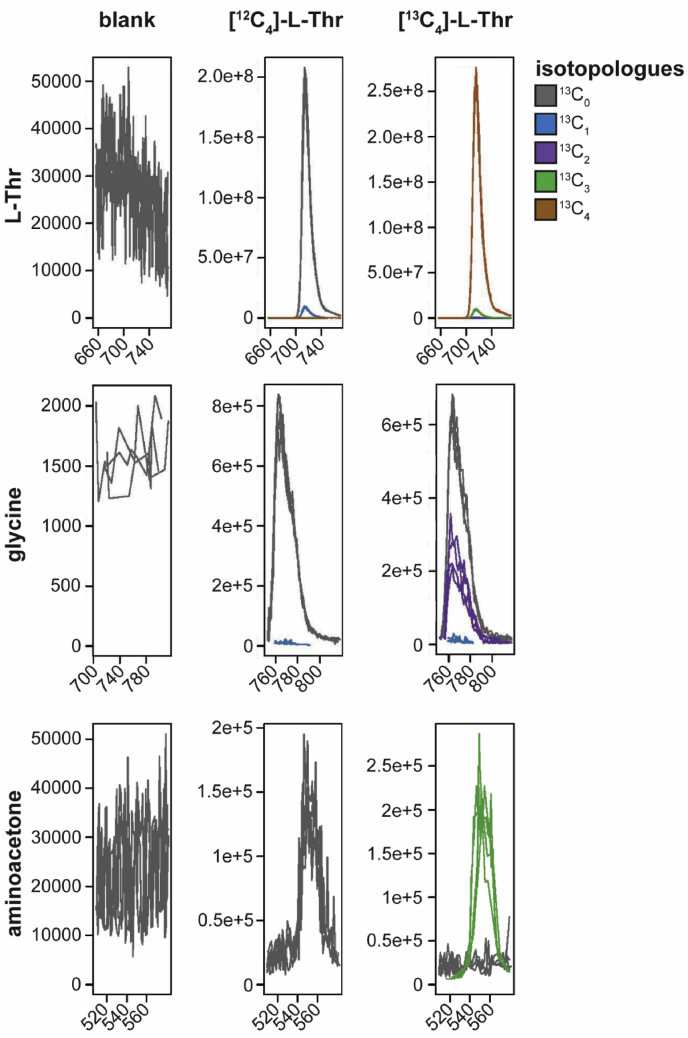
Results of the [U-^13^C]-L-Thr f**lux assay.***E. multilocularis* metacestode vesicles were cultured with 5 mM [U-^13^C]-L-Thr or unlabeled L-Thr for 24 h at 37 °C under a humid, microaerobic atmosphere (n = 4). Shown are graphs with for the metabolites L-Thr, glycine and aminoacetone in vesicle medium (VM) via LC-MS. Raw area under the curve (AUC) values and fractional enrichment for each isotopologue are shown in S3 Table.

### 3.3 Genes for Thr catabolism are present and expressed in the *E. multilocularis* metacestode stage

In order to search for Thr metabolism genes within *E. multilocularis,* we blasted protein sequences of TD, TDH, KBL and TA from various reference organisms (*C. elegans, D. rerio, D. melanogaster*, *H. sapiens* and *M. musculus*) against the protein database of *E. multilocularis*. TD sequences from *H. sapiens* and *M. musculus* both resulted in one hit (EmuJ_001093200). TD sequences from *C. elegans* and *D. melanogaster* did not identify hits. TDH sequences from *C. elegans, D. rerio*, *D. melanogaster* and *M. musculus* found only one hit (EmuJ_000511900). KBL sequences of *C. elegans*, *D. rerio, D. melanogaster*, *H. sapiens* and *M. musculus* found several hits, but EmuJ_000107200 corresponded to the lowest E-values in all blasts. None of the tested TA sequences from *C. elegans, D. rerio*, *D. melanogaster* and *M. musculus* resulted in any hits within the protein database of *E. multilocularis*. Reciprocal blasts of EmuJ_001093200, EmuJ_000511900 and EmuJ_000107200 were performed against protein databases of *C. elegans, D. rerio*, *D. melanogaster, H. sapiens* and *M. musculus*. Reciprocal blasts confirmed EmuJ_001093200 as EmTD, EmuJ_000511900 as EmTDH and EmuJ_000107200 as EmKBL. Accession numbers and results of the BLASTP and reciprocal BLASTP with all sequences producing significant alignments and E-values can be found in S4 Table, S5 Table and S6 Table. We further performed BLASTP with TDH sequences from the same reference organisms against the protein databases of the closely related parasite *E. granulosus s.s.* (S7 Table) and subsequent reciprocal blasts confirmed EgrG_000511900 as EgTDH (S8 Table). An amino acid alignment is shown in S4 Fig.

We then analyzed gene expression of *emtd*, *emtdh* and *emkbl* relative to the house keeping gene *emelp in vitro* under axenic, microaerobic culture conditions (Table 1) and found that both *emtdh* (relative expression of 0.55 ± 0.04) and *emkbl* (relative expression of 1.32 ± 0.26) were higher expressed than *emtd* (relative expression of 0.002 ± 0.0003). We repeated the experiment once and came to the same conclusion (Table 1). We also analyzed the expression of these genes in metacestode tissue obtained from experimentally infected mice and again noted higher expression of *emtdh* (relative expression of 0.61 ± 0.09) and *emkbl* (relative expression of 0.33 ± 0.05) compared to *emtd* (relative expression of 0.0004 ± 0.0002) (Table 1).

**Table 1:** **mRNA levels and protein abundances of three*****E. multilocularis*****genes involved in Thr catabolism, as well as the housekeeping gene*****emelp*****in tissue of the metacestode stage.** Relative expression was analyzed in the *E. multilocularis* metacestode stage for metacestode vesicles cultured *in vitro* metacestode tissue obtained from experimentally infected mice (n = 4 for both). q-RT-PCRs were performed in technical duplicates for each sample and gene expression was calculated relative to the housekeeping gene *emelp*. Two independent experiments were performed to analyze gene expression *in vitro* and one experiment was performed to analyze gene expression *ex vivo*. The protein abundances given as iBAQ values were determined in tissue from *in vitro* cultured metacestode vesicles as previously published (see Müller et al. (2023) for the complete dataset). N.D. = not detected.

Gene	accession N°	*in vitro* I	*in vitro* II	*ex vivo*	Protein abundance (iBAQ∗10^9^)
*emelp*	EmuJ_000485800	1.01 ± 0.14	1.01 ± 0.15	1.01 ± 0.14	3.0 ± 0.17
*emtd*	EmuJ_001093200	0.002 ± 0.0003	0.003 ± 0.001	0.0005 ± 0.0002	N.D.
*emtdh*	EmuJ_000511900	0.55 ± 0.04	0.96 ± 0.23	0.61 ± 0.09	0.96 ± 0.08
*emkbl*	EmuJ_000107200	1.32 ± 0.26	3.79 ± 1.08	0.33 ± 0.05	2.66 ± 0.23

In a recent study, EmTDH and EmKBL were found via non-targeted proteomics of VF and VT of *in vitro* cultured metacestode vesicles, as well as in VF of *in vivo* grown metacestodes obtained from experimentally infected mice (Müller et al., 2023). Further confirming our expression analysis, EmTD was not detected in these samples.

### 3.4 The protein EmTDH is expressed and enzymatically active in the *E. multilocularis* metacestode stage

To assess whether *emtdh* is translated into an enzymatically active protein in *E. multilocularis in vitro*, we measured enzymatic activity of EmTDH in crude extracts of *in vitro* cultured metacestode vesicles. We observed a dose-dependent increase in absorbance, indicating enzyme activity upon addition of the substrate L-Thr, but not D-Thr (Fig. 4A).

**Fig. 4:**
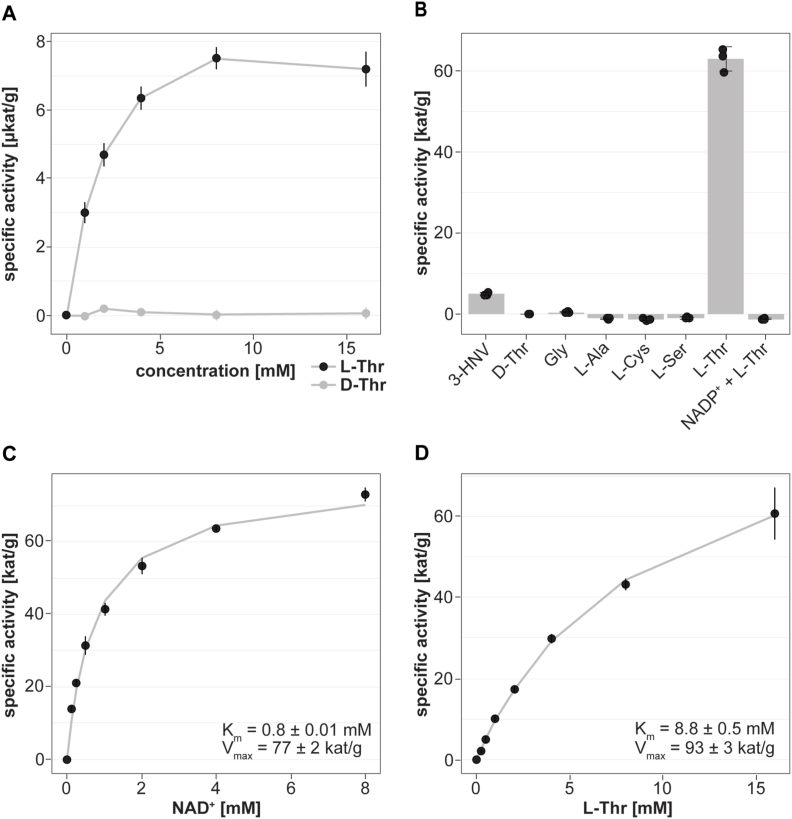
Functional TDH assays with *E. multilocularis* metacestode vesicle tissue crude extracts and recombinant EmTDH. Enzymatic assays were performed by measuring the increase in NADH formation via the increase in absorbance at 340 nm at 37 °C in technical triplicates. A, *E. multilocularis* metacestode vesicle tissue crude extract (80 μg protein per well) with 10 mM NAD^+^ and varying concentrations of L-Thr or D-Thr (0, 1, 2, 4, 8 and 16 mM). B, recEmTDH (0.05 μg protein per well) was tested with 10 mM NAD^+^ in combination with various amino acids (3-HNV, D-threonine (D-thr), glycine (gly), L-alanine (L-Ala), L-cysteine (L-Cys), L-serine (L-Ser), L-threonine (L-Thr)) and L-Thr with NADP^+^. C, recEmTDH (0.05 μg protein per well) was tested with varying concentrations of NAD^+^ (0, 0.125, 0.25, 0.5, 1, 2, 4 and 8 mM) and 16 mM L-Thr. Grey line shows the fitted Michaelis-Menten curve. D, recEmTDH (0.05 μg protein per well) was tested with varying concentrations of L-Thr (0, 0.25, 0.5, 1, 2, 4, 8 and 16 mM) and 2 mM NAD^+^. Grey line shows the fitted Michaelis-Menten equation.

We recombinantly expressed EmTDH (S5 File) and also here detected L-Thr-dependent activity (Fig. 4B). We also evaluated specificity of recEmTDH to NAD^+^ and detected no recEmTDH activity upon incubation with NADP^+^ as well as different amino acids including glycine, L-alanine, L-cysteine, L-serine, only fractional activity was observed with the L-Thr analogue 3-HNV. We further performed kinetic studies evaluating recEmTDH activity with various concentrations of NAD^+^ (Fig. 4C) or L-Thr (Fig. 4D). The calculated K_m_ values were 8.8 ± 0.05 mM for L-Thr and 0.8 ± 0.01 mM for NAD^+^, respectively. The maximal velocity of recEmTDH was 93 ± 3 kat/g corresponding to 313 ± 16 s^−1^ for the titration of L-Thr and 77 ± 2 kat/g corresponding to 257 ± 5 s^−1^ for the titration of NAD^+^.

As a control protein, we recombinantly expressed MmTDH (S5 File) and this protein was also enzymatically active (S5 Fig).

### 3.5 EmTDH can be slightly inhibited by repurposed TDH inhibitors

We applied the enzymatic assays for recEmTDH and recMmTDH to test the activities of various previously published TDH inhibitors (disulfiram, myricetin, quercetin, seven QCs and sanguinarine) (Adjogatse, 2015; Alexander et al., 2011; Cross et al., 1975) (Fig. 5).

**Fig. 5:**
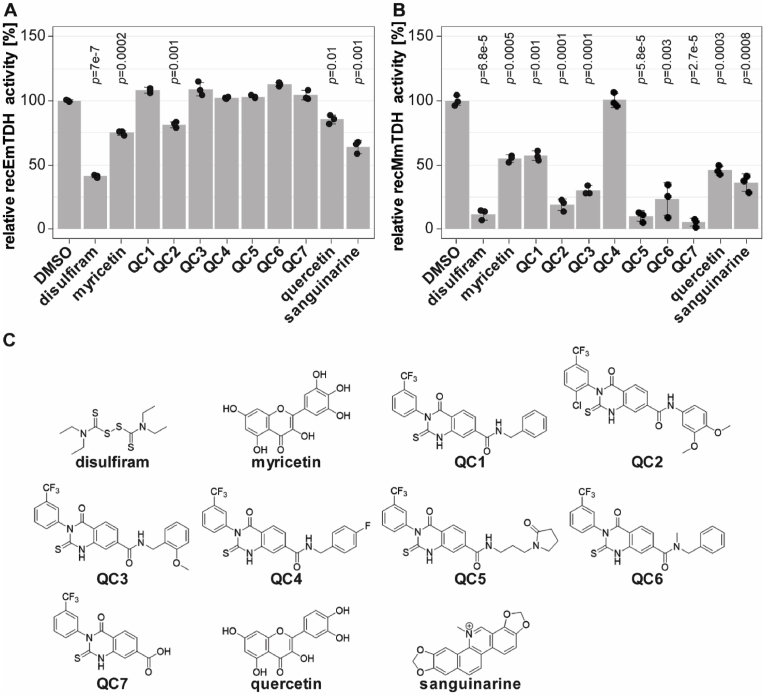
Effect of various potential TDH inhibitors on recEmTDH and recMmTDH and structures of potential TDH inhibitors. TDH activity was assessed as in Fig. 4. Compounds were tested at 20 μM and shown are mean values and SDs of three technical replicates. Shown are relative activities of recEmTDH (A) or recMmTDH (B) treated with different compounds at 20 μM compared to their respective DMSO control with Bonferroni-corrected *p-*values. C, structures of potential TDH inhibitors tested against recEmTDH and recMmTDH in this study. The synthesis for QC1 to QC7 is described in S1 File.

In relation to the DMSO control, several compounds, when applied at 20 μM, slightly, but significantly reduced the activity of recEmTDH. Enzymatic activity was reduced to 41.2 ± 0.8% for disulfiram (*p* = 7e-7), 74.8 ± 1.7% for myricetin (*p* = 0.0002), 81.3 ± 1.8% for QC2 (*p* = 0.001) 85.5 ± 2.8% for quercetin (*p* = 0.01) and 64.0 ± 4.0% (*p* = 0.001) for sanguinarine. All tested compounds, except QC4, inhibited recMmTDH activity, exhibiting stronger inhibitory effects compared to recEmTDH.

### 3.6 Sanguinarine is active against *E. multilocularis in vitro*

**Fig. 6:**
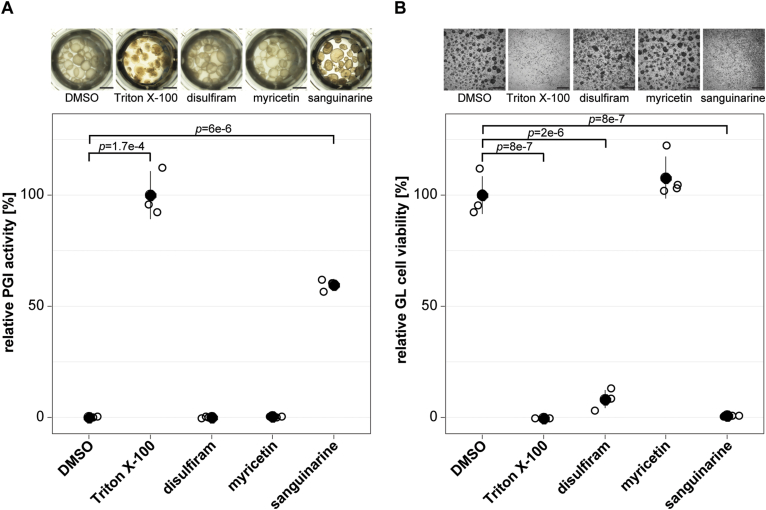
Effect of recEmTDH inhibitors on *E. multilocularis* metacestode vesicles and GL cell cultures. A, *E. multilocularis* metacestode vesicles were incubated with the negative control 0.1% DMSO, the positive control 0.1% Triton X-100, or the three recEmTDH inhibitors disulfiram, myricetin and sanguinarine at 20 μM (n = 3 for each condition). Metacestode vesicles were incubated under a humid, microaerobic atmosphere and after five days, pictures were taken and damage marker release was assessed relative to Triton X-100 treatment. Shown are photos of metacestode vesicles upon treatment (scale bars = 2 mm), as well as PGI assay results with individual values (empty circles), mean values (filled circles), SDs and Bonferroni-corrected *p*-values of one representative experiment. The second independent experiment is shown in S6 Fig. B, *E. multilocularis* GL cell cultures were incubated with recEmTDH inhibitors disulfiram, myricetin and sanguinarine at 20 μM, the negative control 0.2% DMSO and the positive control 0.1% Triton X-100, (n = 4 for each condition). GL cell cultures were incubated under a humid, microaerobic atmosphere. After five days, pictures were taken (scale bars = 200 μm), and cell viability was measured and calculated relative to DMSO. Shown are individual values (empty circles) mean values (filled circles), SDs and Bonferroni-corrected *p*-values of one representative experiment. The other experiment is shown in S6 Fig.

The three most active recEmTDH inhibitors disulfiram, myricetin and sanguinarine were not specifically active against recEmTDH over recMmTDH and certainly future drug screens should aim at identifying more potent and specific inhibitors. However, we here tested the activity of these three moderate inhibitors on *E. multilocularis* metacestode vesicles via damage marker release assay (PGI assay) and GL cell viability assay (Fig. 6). The negative control DMSO did not affect the physical appearance of metacestode vesicles after five days (Fig. 6A), while the internal control Triton X-100 led to maximum physical damage and PGI release (100 ± 8.8 %, *p* = 1.7e-4). Disulfiram and myricetin did not affect metacestode vesicle integrity and showed no activity in the PGI assay (−0.2 ± 0.3 % and 0.1 ± 0.1 %, respectively). On the other hand, metacestode vesicles treated with sanguinarine all collapsed after five days of *in vitro* treatment, and strong activity was shown in the PGI assay (59.5 ± 2.2%, *p* = 6e-6). We repeated this experiment once independently and obtained similar results with significant activity of sanguinarine against metacestode vesicles (S6 Fig). In the GL cell viability assay, DMSO resulted in the formation of round aggregates with a GL cell viability of 100 ± 7.4%. No aggregates were observed at all upon treatment with the positive control Triton X-100 and GL cell viability was highly reduced (−0.4 ± 0%, *p* = 8e-7) (Fig. 6B). Treatment of GL cell cultures with disulfiram resulted in irregularly shaped aggregates and strongly reduced GL cell viability (8 ± 3.5%, *p* = 2e-6) while myricetin had no effect on aggregate formation nor on GL cell viability (107.8 ± 8.3%). No aggregates were formed upon treatment with sanguinarine, and GL cell viability was strongly reduced (0.5 ± 0.1%, *p* = 8e-7). We repeated this experiment once independently and obtained similar results with significant activity of disulfiram and sanguinarine against GL cells (S6 Fig).

## 4 Discussion

AE is a severe zoonotic disease with limited curative treatment options (Brunetti et al., 2010). New drugs are urgently needed and a better understanding of the metabolism may lead to the discovery of novel targets for interventions (Geary, 2012; Lundström-Stadelmann et al., 2019). Previously, our group has reported on the high uptake of the amino acid L-Thr by *E. multilocularis* metacestode vesicles *in vitro* (Ritler et al., 2019) and uptake of L-Thr was also reported from *E. granulosus s.l.* metacestodes *ex vivo* obtained from experimentally infected mice (Jeffs and Arme, 1988). Thr as a source for energy generation has been suggested to play a role in various parasites such as *Entamoeba* spp. (Zuo and Coombs, 1995)*, T. brucei* (Cross et al., 1975; Millerioux et al., 2013) and *Trichomonas vaginalis* (Zuo et al., 1995). In this project, we analyzed which effects L-Thr has on *E. multilocularis in vitro*, how L-Thr is metabolized and if the Thr-catabolic pathways would suggest potential targets for new interventions.

Growth assays provide simple and efficient tools to study the effects of nutrients on larval parasites. Different forms of measuring growth of *E. multilocularis* metacestode vesicles have been applied in the past, such as measuring their diameter via a microscope (Cheng et al., 2017) or via volume measurements of single or multiple metacestode vesicles in falcon tubes (Förster et al., 2019; Spiliotis et al., 2004; Spiliotis and Brehm, 2009). These assays provided valuable information regarding the improvement of *in vitro* culture conditions and the effects of growth factors on metacestode vesicles. However, when evaluating the effect of nutrients on single metacestode vesicles combined with an upscaling of replica, these measurement methods are highly time-consuming. Here, we established a growth assay using robust numbers of metacestode vesicles per condition (24 replica) in which the metacestode vesicle diameter is analyzed via automated and semi-automated scripts in ImageJ, enabling a fast and objective analysis. Both scripts reliably measured the diameter of metacestode vesicles faster and with a smaller error than the manual measurements. We thus employed these scripts to assess metacestode vesicle growth, which allowed us to use large numbers of replica. Our results showed that growth of *E. multilocularis* metacestode vesicles and development of metacestode vesicles from GL cell cultures was dependent on an active Thr metabolism, where L-Thr addition showed positive effects and its non-proteinogenic analogue 3-HNV negative effects. This could be explained by a possible competition of L-Thr and 3-HNV as TDH substrates, as suggested for TDH from mouse embryonic stem cells (Wang et al., 2009). This process also seemed to occur within *E. multilocularis* metacestode vesicles, as a combination of L-Thr and 3-HNV counteracted the negative effect of 3-HNV alone. We thus could show that in a standardized *in vitro* setting (Müller et al., 2023), an active L-Thr metabolism is important for *E. multilocularis*. It is of note that we encountered variation in the number of metacestode vesicles formed from GL cell cultures, even in the control groups between different assays. These batch-effects were probably caused by the isolation and cultivation of varying numbers of GL cells, which currently cannot be standardized in a better way.

To investigate which L-Thr catabolism pathways are active in *E. multilocularis in vitro*, we performed a [U-^13^C]-L-Thr flux assay that showed uptake of L-Thr by metacestode vesicles, metabolization to glycine and subsequent secretion of this metabolite to the culture medium, thus confirming previous reports (Ritler et al., 2019). The presence of [U-^13^C]-aminoacetone and [U-^13^C]-glycine, as well as lack of detection of α-ketobutyrate in our metabolomic samples, suggests an EmTDH-mediated L-Thr catabolism. In *C. elegans*, the acetyl-coenzyme A that is generated together with glycine via KBL feeds into the TCA cycle (Yilmaz and Walhout, 2016). Acetyl-coenzyme A was not detected in our samples, as the here applied detection method was not optimized for this metabolite. We detected a variety of TCA cycle intermediates, but none of them contained L-Thr-derived [^13^C]. This indicates that at least within the 24 h of our *in vitro* setup under microaerobic conditions, L-Thr uptake did not feed into the TCA cycle. The protozoan parasite *T. brucei* also was reported to metabolize L-Thr via TDH and KBL and the generated acetyl-coenzyme A did not majorly feed into the TCA cycle, but rather was used for the synthesis of acetate and lipids (Cross et al., 1975; Gilbert et al., 1983; Klein and Linstead, 1976; Linstead et al., 1977; van Weelden et al., 2003). We did not detect acetate and although we detected propionate and palmitate in our samples, they did not incorporate [^13^C]. However, given that these metabolites were also present in the blank samples, we cannot exclude that a small proportion contained [^13^C] that was masked by the blank samples.

L-Thr has been shown to be essential for cell proliferation and DNA synthesis of mouse embryonic stem cells (Wang et al., 2009). L-Thr is hereby metabolized via TDH to glycine and acetyl-coenzyme A, which is used for the synthesis of *S*-adenosylmethionine (SAM) (Shyh-Chang et al., 2013). Restriction of L-Thr from *in vitro* cultured mouse embryonic stem cells decreased trimethylation of histone H3 lysine 4 (Shyh-Chang et al., 2013). SAM was not detected in our set up, but its precursor L-methionine did not incorporate [^13^C]. However, potential effects of L-Thr on the histone modification in *E. multilocularis* metacestode vesicles were not further focus of our study.

Besides the results of the [U-^13^C]-L-Thr flux assay, we also measured significantly higher gene expression of *emtdh* and *emkbl* compared to *emtd* in metacestode vesicles, confirming previously reported results in metacestode vesicles *in vitro* and metacestodes *in vivo* (Huang et al., 2017; Tsai et al., 2013). Furthermore, EmTDH and EmKBL, but not EmTD, were detected via non-targeted proteomics in VF and VT of *in vitro* cultured metacestode vesicles and also in VF of *in vivo* grown metacestodes obtained from experimentally infected mice (Müller et al., 2023). Finally, by testing crude extracts of *in vitro* cultured *E. multilocularis* metacestode vesicles in an enzymatic assay, we confirmed that EmTDH was translated into an enzymatically active protein. Taken together, our experiments strongly suggest that EmTDH is the major L-Thr catabolic enzyme in *in vitro* cultured metacestode vesicles. Given that L-Thr did not feed into the TCA cycle in our [U-^13^C]-L-Thr flux assay, energy generation via the TCA cycle cannot explain the positive effects of L-Thr on metacestode vesicle growth and development.

Since our experiments confirmed the relevance of a TDH-mediated L-Thr metabolism in *E. multilocularis in vitro*, we wanted to investigate whether this enzyme could serve as a potential drug target candidate. We recombinantly expressed MmTDH alongside since human TDH is a non-functional pseudogene (Edgar, 2002) and potential selective recEmTDH inhibitors would be first tested in the mouse models of AE (Hinz, 1973; Ohbayashi, 1960).

We tested a series of QCs, which have been found active against recMmTDH upon a high-throughput screen of 20,000 compounds (Alexander et al., 2011) and additionally investigated the effects of disulfiram, myricetin, quercetin and sanguinarine, which have been reported to show activity against recTbTDH from *T. brucei* (Adjogatse, 2015). Disulfiram, myricetin and sanguinarine partially, but not specifically, inhibited EmTDH activity. However, when tested on *E. multilocularis* metacestode vesicles, only sanguinarine displayed notable anti-parasitic effects.

In the study presented here, we identified sanguinarine to be active against recEmTDH, as well as metacestode vesicles and isolated GL cells *in* vitro. Further studies are necessary to evaluate additional cellular targets of sanguinarine in *E. multilocularis*. As an alkaloid, sanguinarine has been reported to inhibit multiple targets including Na^+^ K^+^-ATPase, acetylcholine esterase, butyrylcholinesterase, choline acetyl transferase, as well as protein kinases. (Mackraj et al., 2008). Due to its high toxicity (Babich et al., 1996; Vrba et al., 2009) and only moderate activity, sanguinarine is unlikely to be developed further as anti-echinococcal drug. However, sanguinarine served as a proof-of-concept to demonstrate that recEmTDH inhibitors can have activity against *E. multilocularis.* It may serve as a basis for designing derivatives with improved specificity and potency against the parasite.

Sanguinarine had previously been reported to perturb anterior regeneration of the planarian *Dugesia japonica* (Balestrini et al., 2017) and to be active against the ciliate *Ichthyophthirius multifiliis in vitro* and in an *in vivo* grass carp (*Ctenopharyngodon idella*) model (Yao et al., 2010). Furthermore, sanguinarine demonstrated activity against the nematode *Toxocara canis in vitro* (Satou et al., 2002) and *Trichinella spiralis in vitro* and in an *in vivo* mouse model (Huang et al., 2020). Regarding parasitic platyhelminths, reported effects of sanguinarine include activity against the monogenean parasite *Dactylogyrus intermedius* in *in vivo* models with goldfish (*Carassius auratus*) (Wang et al., 2010; Zhang et al., 2013), the trematode *Schistosoma mansoni in vitro* (Lalli et al., 2015; Zhang and Coultas, 2012) and *in vitro* cultured protoscoleces of the cestode *E. granulosus sensu lato* (Hassanzadeh et al., 2023).

In future it will be crucial to evaluate the relevance of EmTDH-mediated L-Thr metabolism for *E. multilocularis in vivo* and further confirm EmTDH as a potential drug target. Upon its validation, the here established enzymatic assay for EmTDH could serve as a discovery platform that allows for targeted medium-throughput screening of inhibitors. It should be further investigated whether this tool could also be used to screen potential inhibitors against TDH of the closely related *E. granulosus s.s.* (EgTDH: EgrG_000511900, PRJEB121), which is the causative agents for human cystic echinococcosis. Active and specific drugs could then be confirmed via the here mentioned whole-organism-based *in vitro* screening assays, as they have been recently validated for *E. granulosus s.s.* (Kaethner et al., 2023).

## CRediT authorship contribution statement

**Marc Kaethner:** Writing – review & editing, Writing – original draft, Visualization, Validation, Software, Project administration, Methodology, Investigation, Formal analysis, Data curation, Conceptualization. **Pascal Zumstein:** Writing – review & editing, Validation, Methodology, Investigation, Formal analysis. **Joachim Müller:** Conceptualization, Data curation, Formal analysis, Methodology, Writing – review & editing. **Matías Preza:** Writing – review & editing, Validation, Investigation. **Philipp Grossenbacher:** Writing – review & editing, Visualization, Methodology. **Anissa Bartetzko:** Writing – review & editing, Validation, Investigation. **Laura Vetter:** Methodology, Writing – review & editing. **Martin Lochner:** Writing – review & editing, Conceptualization. **Stefan Schürch:** Validation, Methodology, Investigation, Conceptualization. **Clement Regnault:** Writing – review & editing, Validation, Methodology, Investigation, Formal analysis, Data curation. **Daniel Villalobos Ramírez:** Writing – review & editing, Software, Methodology. **Britta Lundström-Stadelmann:** Writing – review & editing, Writing – original draft, Visualization, Validation, Supervision, Software, Project administration, Methodology, Investigation, Funding acquisition, Formal analysis, Data curation, Conceptualization.

## Funding statement

Swiss National Science Foundation, grant nb. 192072 to BLS. The funders had no role in study design, data collection, analysis and interpretation, decision to publish, or preparation of the manuscript.

## Figures and Tables

**figs1:**
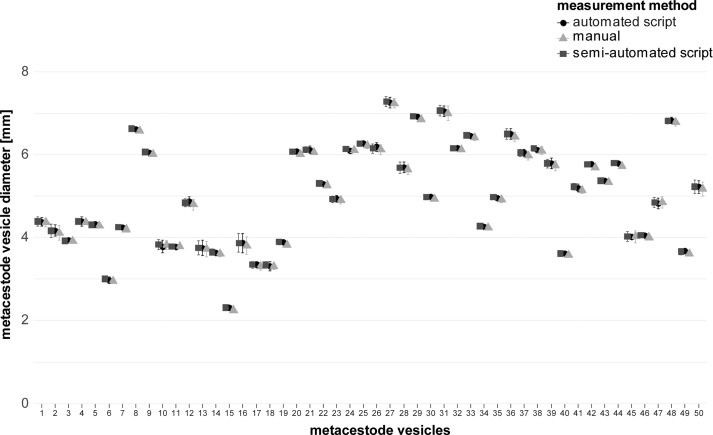


**figs2:**
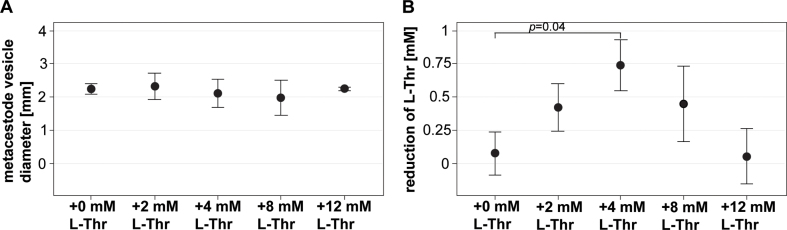


**figs3:**
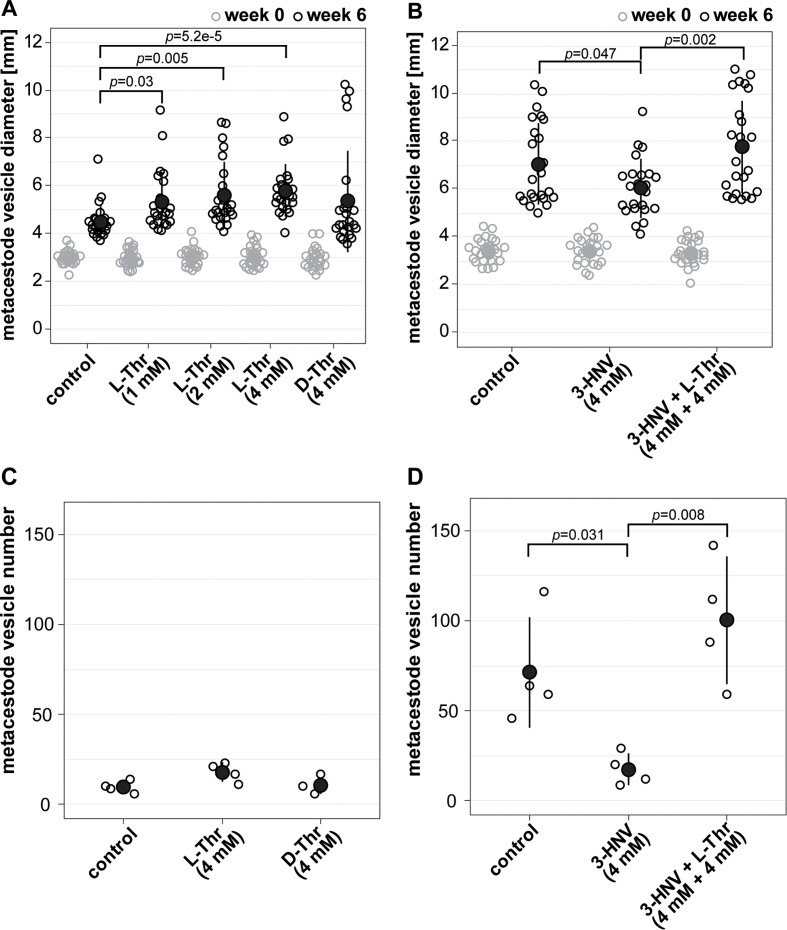


**figs4:**
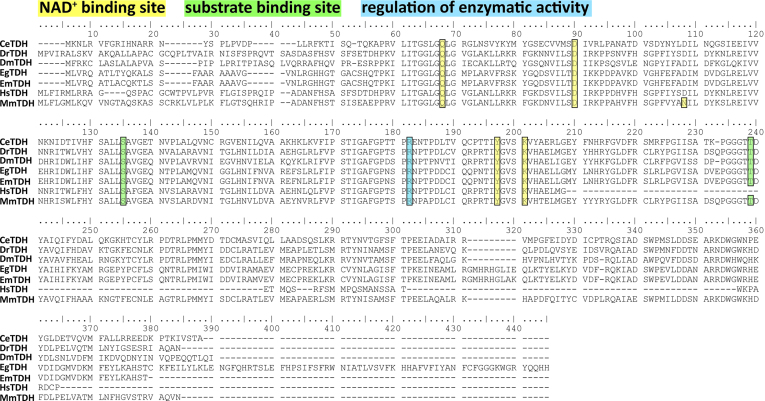


**figs5:**
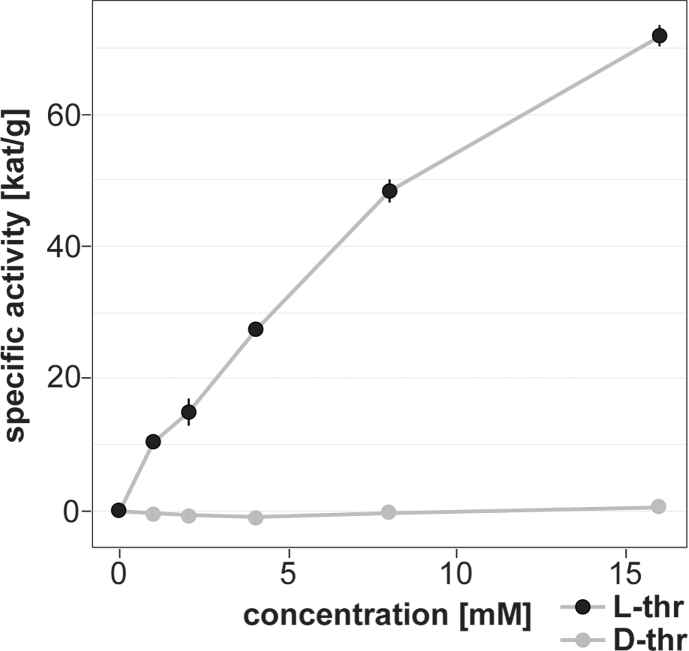


**figs6:**